# Tandem Androgenic and Psychological Shifts in Male Reproductive Effort Following a Manipulated “Win” or “Loss” in a Sporting Competition

**DOI:** 10.1007/s12110-018-9323-5

**Published:** 2018-08-09

**Authors:** Daniel P. Longman, Michele K. Surbey, Jay T. Stock, Jonathan C. K. Wells

**Affiliations:** 1 Department of Archaeology and Anthropology, University of Cambridge, Cambridge, CB2 3QG UK; 2 Department of Psychology, James Cook University, Townsville, QLD 4811 Australia; 3 Department of Anthropology, University of Western Ontario, London, Ontario Canada; 4 Childhood Nutrition Research Centre, UCL Institute of Child Health, London, WC1N 1EH UK

**Keywords:** Reproductive effort, Trade-off, Testosterone, Mating effort, Parenting effort

## Abstract

Male-male competition is involved in inter- and intrasexual selection, with both endocrine and psychological factors presumably contributing to reproductive success in human males. We examined relationships among men’s naturally occurring testosterone, their self-perceived mate value (SPMV), self-esteem, sociosexuality, and expected likelihood of approaching attractive women versus situations leading to child involvement. We then monitored changes in these measures in male rowers (*N* = 38) from Cambridge, UK, following a manipulated “win” or “loss” as a result of an indoor rowing contest. Baseline results revealed that men with heightened testosterone and SPMV values typically had greater inclinations toward engaging in casual sexual relationships and a higher likelihood of approaching attractive women in a hypothetical social situation. As anticipated, both testosterone and SPMV increased following a manipulated “victory” and were associated with heightened sociosexuality, and increased expectations toward approaching attractive women versus individuals who would involve them in interacting with children after the race. SPMV and self-esteem appeared to mediate some of the effects of testosterone on post-race values. These findings are considered in the broader context of individual trade-offs between mating and parental effort and a model of the concurrent and dynamic androgenic and psychological influences contributing to male reproductive effort and success.

**Electronic supplementary material:**

The online version of this article (10.1007/s12110-018-9323-5) contains supplementary material, which is available to authorized users.

## The Mediation of Reproductive Success by Status and Testosterone

In many animal populations, status in a social hierarchy is positively correlated with reproductive success (Ellis 1995; Strier 2003). Greater reproductive success may result from increased access to resources, decreased harassment by other group members, and the reduced risk of predation inherent in enhanced social status. In both sexes, this promotes reduced emotional stress and better health, leading to greater chances of copulation, conception, and birth of healthy offspring (Ellis 1995). This trend is evident in both preindustrial and contemporary Western human societies. Male status within communities such as the !Kung of the Kalahari and the Aché of Paraguay relates positively to number of surviving offspring, as does income in the contemporary United States and Britain (Buss 1989; Hopcroft 2006; Kaplan and Hill 1985; Nettle and Pollet 2008; Pennington and Harpending 1993).

Status is often determined by male-male competition (Altmann et al. 1995). Across a range of mammalian species, testosterone closely tracks the results of such dominance interactions (Zilioli and Watson 2012). Furthermore, increased testosterone levels may encourage dominant behavior intended to achieve or maintain high status. The changes in androgenization caused by competition may have consequences for future status-seeking behavior, as proposed by the reciprocal model of testosterone and status (Mazur and Booth 1998). As such, testosterone both affects and is affected by status-seeking competition. The reciprocal model may therefore illuminate both winning and losing streaks (Booth et al. 1989).

Among human males, testosterone levels generally show a relative increase following victory compared with defeat in both athletic and non-athletic contests, as observed in tennis, wrestling, chess, timed reaction tests, monetary competitions, tasks of chance, and the vicarious experience of winning amongst sports fans (Apicella et al. 2014; Archer 2006; Bernhardt and Dabbs 1997; Bernhardt et al. 1998; Booth et al. 1989; Elias 1981; Gladue et al. 1989; Mazur and Lamb 1980; Mazur et al. 1992; McCaul et al. 1992). Similar findings have recently been reported in non-industrialized human populations with respect to hunting success (Trumble et al. 2013) and in primates following a dominance interaction (Muller and Wrangham 2001). This relationship is not surprising since competitions have been described as formalized contests for status in which winners enjoy a status gain relative to losers (Edwards 2006). However, several investigations report nonsignificant differences in testosterone levels between winners and losers in both individual and team sports as well as video game contests (Gonzalez-Bono et al. 2000; Mazur et al. 1997; Salvador et al. 1987, 1990; Suay et al. 1999).

### The Role of Testosterone in Male Mating Effort

According to the Immunocompetence Handicap Hypothesis (Folstad and Karter 1992), which builds upon Zahavi’s (1975) handicap hypothesis for the evolution of secondary sexual characteristics, testosterone mediates signals of underlying reproductive status and quality in humans. Male muscle mass, whose synthesis is enhanced by testosterone, may serve as such a signal in humans (Griggs et al. 1989; Kadi 2008). Muscular men are more sexually attractive than slender men and report more sexual partners as well as a younger age at first intercourse (Frederick and Haselton 2007; Gallup et al. 2007; Lavrakas 1975). Muscularity is advantageous in male intrasexual competition, as well as in provoking jealousy in male rivals (Bribiescas 2001; Dijkstra and Buunk 2002; Frederick and Haselton 2007). Testosterone therefore plays an important moderating role in the physiological aspects of male reproductive effort through increasing chances of successful male-male competition and also in attracting females.

Any signal that is expensive to construct and maintain from an energetic or otherwise fitness-related perspective will serve as an indicator of male quality (Andersson 1994). Selection may then favor the opposite sex being attracted to such signals (Graffen 1990; Zahavi 1975). Skeletal muscle mass is an expensive tissue to maintain, accounting for approximately 20% of human male basal metabolic rate (Elias 1992). Further burden is placed on the male through testosterone-induced suppression of the immune system (Folstad and Karter 1992). Elevated levels of testosterone have been linked to increased incidence of prostate cancer, oxygen radical production, reduced tissue and organ maintenance, and injury associated with aggressive confrontational behavior (see Lassek and Gaulin 2009; Muehlenbein 2006). The high energetic demands associated with muscle mass mean that during periods of energetic deficit there may be a suppression of testosterone levels. This leads to reduced somatic reproductive effort by decreasing muscle mass (Bribiescas 2001). However, although there is evidence for a direct link between testosterone and male musculature in nonhuman primates, similar evidence in humans is considered weak (Alvarado et al. 2015). Alvarado et al. (2015) present evidence suggesting that muscle mass and strength are enhanced by fatherhood, despite decreased testosterone levels. This “paternal provisioning hypothesis” predicts that men’s skeletal muscle mass is less dependent on testosterone than it is for nonhuman primates.

Testosterone has also been implicated in the behavioral aspects of male mating effort (see Roney and Gettler’s 2016 review of the role of testosterone in mating effort). Underscoring the motivational role of male sexual desire, Darwin noted that “males. . . are almost always the more active and most often, the initiators of sexual interactions” (Darwin 1871). Although sexual desire, or libido, is a concept that has evaded precise definition (Bancroft 1988), many studies have attempted to understand how it is generated. Despite the literature being somewhat mixed, and recent work suggesting no significant association between testosterone and desire in men (Van Anders 2012), androgens appear to play an important role in its production (Baumeister et al. 2001). In addition to impacting sexual motivation when a threshold is reached (Bagatell et al. 1994), androgens regulate the competitive behavioral components of male mating effort as well as mediating confidence and assertiveness in social situations—qualities deemed to be beneficial in male mating effort (Bagatell et al. 1993; Ellison 2003; Morley 2003). Consequently, higher levels of testosterone have been found to be associated with a greater number of sexual partners (Bogaert and Fisher 1995) and mating success (defined as the number of sexual partners an individual has had; Peters et al. 2008). Furthermore, men who are single or have multiple sexual partners have been shown to have higher testosterone levels than men in committed monogamous relationships (McIntyre et al. 2006; Van Anders et al. 2007; Van Anders and Watson 2007). Therefore, in addition to influencing libido, high testosterone levels seem to facilitate heightened competition for female mates, the adoption of short-term strategies, and the subsequent pursuit of multiple mates. The present study aims to build on previous work (van der Meij et al. 2012), which demonstrated that elevated testosterone levels following male-male competition may be followed by increased affiliative behavior toward women, such as increased smiling and eye contact. This will enhance understanding of whether human short-term fluctuations in testosterone reveal analogous behavioral effects of male mating effort at the intraindividual level.

### Variations in Testosterone and Parenting Effort

Life history theory predicts trade-offs in energetic investment in key physiological processes such as reproduction, maintenance, and growth (Charnov 1993; Roff 1992; Stearns 1992). Reproductive effort is the sum of mating (time and energy invested in attraction, pursuing mates, and competition) and parental effort (all forms of energy and time invested in offspring). The trade-off between mating and parental effort may be one of the most common life history trade-offs (McGlothlin et al. 2007). In addition to varying across species, the outcome of this trade-off may also vary within species and individuals depending on age and environmental conditions. In species with biparental care, such as humans and birds, males confront a trade-off between the acquisition of mates and investment in offspring since these activities compete for time and energy given limited budgets. In these species, variations in testosterone levels between males appear to reflect different allocations of mating versus parental effort.

There is convincing evidence that testosterone levels are reduced in men who are in love, married, or otherwise pair bonded relative to those of single men (Booth and Dabbs 1993; Burnham et al. 2003; Gettler et al. 2011a; Gray et al. 2002, 2004a, 2007a; Marazziti and Canale 2004; Mazur and Michalek 1998; McIntyre et al. 2006). In addition, studies in a variety of human populations show that the transition to, or the attainment of, fatherhood is marked by a decrease in testosterone (although there are exceptions—fathers with older, or more than four, children do not exhibit this reduction in testosterone; e.g., Alvergne et al. 2009; Berg and Wynne-Edwards 2001; Fleming et al. 2002; Gettler et al. 2011b; Gray et al. 2004b, 2006, 2007b; Kuzawa et al. 2009; Muller et al. 2009; Perini et al. 2012; Pollet et al. 2013; Storey et al. 2000).

According to a number of researchers (e.g., Gray et al. 2002; Storey et al. 2000), lower testosterone facilitates paternal caregiving and increased investment in offspring by decreasing competing energetic expenditures in competition and additional mating (which could include extra-pair bonding). In addition to varying according to marital status, testosterone also appears to vary in conjunction with men’s reported ongoing behavioral and emotional investment in parenting effort. Mascaro et al. (2013) found that both testosterone levels and testes volume were negatively correlated with paternal caregiving in fathers, as determined from mothers’ responses on measures of parental responsibility and activity. Similarly, low morning testosterone levels of Senegalese men and low evening levels of testosterone in Filipino men had the highest spouse- and self-report investment in childcare. In addition, Fleming et al. (2002) report that fathers felt more sympathy and experienced a greater need to respond to infant cries if they had lower testosterone levels.

Taken together these findings suggest that the regulation of male reproductive trade-offs through the endocrine system may be a common feature of human populations (Archer 2006). Whether this androgenic regulation is accompanied by complementary psychological changes is much less well studied or established. We might expect, however, that both the physiology and the psychology of human males may reflect facultative trade-offs between mating and parenting effort. Moreover, psychological changes may underlie or mediate the relationship between endocrine function and reproductive behavioral outcomes.

To date, it is unclear whether the transient testosterone surges following victory, which may have implications for male reproductive effort, emerge in conjunction with the physical effort of winning, or as a result of the social perception of winning. An experimental design in which the competition outcome is manipulated would disentangle the two possibilities (similar to the work of Gladue et al. 1989), allowing consideration of the effects of perceived competition outcome separately from the physical act of winning or losing.

### Trade-Offs between Mating and Parental Effort as a Function of Perceived Mate Value

Rationally speaking, the optimal reproductive strategy for human males is to seek multiple shorter-term relationships with reduced investment in offspring (Bateman 1948; Trivers 1972). This is due to the positive relationship between copulation frequency and reproductive success, and the lower level of parental investment minimally required of men (Buss 1989). However, as a result of the heightened requirements imposed by women on casual mates, many men have reduced opportunities to engage in short-term mating strategies (Buss and Schmitt 1993; Gangestad and Simpson 2000). Although the majority of men may have the ability to provide at least some care for children or some level of ongoing resources, fewer have possessed the level of attractiveness to consistently qualify them as short-term mates. Therefore, for many men, the pursuit of a long-term mating strategy with a focus on a single mate and their progeny is their best option. Only those men most attractive to women may be successful in pursuing casual sexual relationships. Therefore trade-offs between mating and parenting effort may vary as a function of men’s SPMV, with higher levels increasing male initiative in seeking partners and mating opportunities versus engaging in relationships associated with investment in children.

Human males and females appear to possess some awareness of their own value as a mate, with one’s self-perceived mate value (SPMV) potentially motivating or mediating mating strategies and effort (Landolt et al. 1995; Surbey and Brice 2007). To test this possibility, Surbey and Brice (2007) experimentally enhanced men’s SPMV resulting in an increase in behavioral intentions to pursue casual over long-term relationships. The attitudinal component of the Sociosexuality Orientation Inventory (SOI, Simpson and Gangestad 1991), a gauge of one’s orientation toward engaging in casual, uncommitted sexual relationships, was similarly heightened. Results further showed that increased SPMV drove these changes in mating strategy rather than a rise in global self-esteem, a psychological characteristic also associated with men’s mating preferences and SPMV (Goodwin et al. 2012; Kiesler and Baral 1970; Surbey and Brice 2007). However, SPMV may be a distinct component of self-esteem especially involved in mating contexts (e.g., Brase and Guy 2004; Kirkpatrick et al. 2002; Webster and Kirkpatrick 2005) and not functionally equivalent to global measures of self-esteem. Moreover, it appears to be a partly inherent, but dynamic, psychological attribute that fluctuates opportunistically in men (e.g., Yong and Li 2012). That SPMV may also be manipulated as a result of male-male competition, or contests between men, including athletic contests typically found to increase testosterone levels in victors, was thus explored.

## Design and Goals of the Current Study

A direct head-to-head rowing ergometer contest, with a manipulated outcome, was employed to construct a situation in which one male participant purportedly defeated the other. Baseline measurements were taken prior to the contest, and repeated after the contest, to determine the effect of competition outcome on salivary testosterone, self-perceived mate value, sociosexuality, global self-esteem, and likelihood of initiating relationships with or approaching attractive women versus individuals associated with investment in children.

In accord with theoretical assumptions (Simpson and Gangestad 1991), men with high baseline testosterone and SPMV were expected to be oriented toward engaging in more casual sexual relationships or exhibit heightened sociosexuality, and display greater intentions to initiate relationships with attractive women. Prior investigations of the relationship between men’s sociosexuality and testosterone have produced mixed results (Charles and Alexander 2011; Farrelly et al. 2015; McIntyre et al. 2006; Puts et al. 2015; Van Anders et al. 2007) and are complicated by such factors as relationship status and variation in the measure of sociosexuality employed. In particular, attitudinal measures appear distinct from behavioral measures (e.g., number of sexual partners), which may be largely influenced by opportunity (Penke and Asendorpf 2008). To reflect this, the Revised Sociosexual Orientation Inventory (SOI-R) was designed to include separate Attitude, Desire, and Behavior facets (Penke and Asendorpf 2008). For example, Edelstein et al. (2011) found significant positive relationships between partnered men’s baseline testosterone levels and scores on the Attitude and Desire, but not Behavior, subscales. Increased baseline SOI-R may be the result of a greater degree of masculinization in utero or subsequently (Puts et al. 2015), but it may also fluctuate according to conditions (Surbey and Brice 2007) and temporary hormonal changes. Testosterone levels and SPMV were expected to increase following a perceived win. These increases were posted to be related and together predict a heightened endorsement of casual sexual relations (indicated by the Attitude or Desire facets of the SOI-R), and greater intentions to approach or pursue attractive women over situations involving caring for or interacting with children. Although self-esteem was expected to vary across some conditions, changes in SPMV were predicted to be the primary psychological predictor of mating intentions and attitudes.

Rowing was chosen because of its physically demanding nature. The strongest correlate of rowing ergometer performance is power output at VO_2 Max_ (Ingham et al. 2002). As such, participants were aware that victory strongly implies the possession of greater physical strength than the opponent, a trait said to be valued by women in choosing a mate (Fink et al. 2007). Furthermore, because it is an individual contest, levels of personal responsibility for competition outcome are high. Previous research has shown that such internal attributions are an important factor in determining the androgen response to victory or defeat (Gonzalez-Bono et al. 1999; Mazur and Lamb 1980). Although all competitors endured the physical act of competing, the link between this effort and competition outcome was removed. Any pursuant physiological or psychological responses would therefore be due to the psychological and social implications of victory in a male-male contest.

## Materials and Methods

### Participants

Forty-two male student rowers (*M* = 20.74 years, *SD* = 1.20, age range = 19–23 years) from the University of Cambridge participated in the study. All participants train for rowing at least five times a week, regularly using and competing on the indoor rowing ergometer. The cohort was deemed homogeneous; all self-reported as being healthy and did not smoke or take drugs or any medication that might influence testosterone levels. During the debriefing, four participants (2 winners, 2 losers) indicated some degree of suspicion regarding the win/loss manipulation and as such, they were removed from the analyses. The analyses were conducted on the remaining 38 participants (19 winners, 19 losers) of which 36 were of European ancestry and 2 of European/Asian heritage. All 38 self-reported as heterosexual. Testing was carried out at a local sporting facility in Cambridge, UK. Ethical approval for the project was granted by the University of Cambridge Human Biology Ethics Committee.

### Procedures and Methods

The experiment consisted of a baseline measuring session and precompetition, competition, and postcompetition phases.

#### Baseline Measuring Session

Participants reported to the laboratory at 1330 h one week prior to racing and completed baseline questionnaires. Questionnaires included demographic and background items and the psychological and behavioral measures described below. To reduce demand characteristics, participants were given only a very general description of the goals of the study, distractor items were included (see below), and baseline data were collected in advance to reduce participants’ recollection of their previous responses or carryover affects in the final phase.

#### Precompetition Phase

On the day of competition, pre-race saliva samples were collected at 1330 h, 30 min prior to the start of the race. All samples were taken at the same time to minimize the effects of diurnal variation in testosterone secretion (Campbell et al. 1982; Touitou and Haus 2000). Participants were instructed not to eat, drink, smoke, or brush their teeth for one hour prior to testing. All samples were immediately chilled and then frozen to −20 °C within an hour of collection.

#### Competition Phase, and Manipulation of Win/Loss Outcome

Each race began at 1400 h. In each race two participants competed for ten minutes on the Concept 2 rowing machine (manufactured by Concept 2, Vermont, USA). The standard machine display was obscured from view, and participants were shown a computer screen displaying only the time remaining and an indicator of who was winning and by how much. The outcome of the race was manipulated, with the winner being selected randomly. The races were designed to be understood to be close and competitive throughout, and the result was clearly displayed on the computer screen immediately upon completion of the race. This manipulation allowed separation of the physical effort from the social implications of winning. Consequently, the mental processing of the result, rather than the actual result, could differentially affect testosterone levels, SPMV, and self-esteem. Participants were matched by their 2000 m personal best (pairs had a difference of 5 s or less). All participants regularly complete this maximum-effort test of fitness, so their personal best is an accurate indicator of their ability on the indoor rowing machine. Participants had a mean 2000 m personal best of 400.29 s (*SD* = 7.42, range 383–415 s).

#### Postcompetition Phase

Post-race questionnaires were administered 10 min after racing, and saliva samples were collected at 1440 h (30 min after racing) (Zilioli and Watson 2013).

### Psychological/Behavioral Measures

#### Self-Perceived Mate Value Questionnaire

The Self-Perceived Mate Value Questionnaire (see ESM for this and other questionnaires) was based on Lalumière and Quinsey’s (1996) integration of previous measures developed by Landolt et al.’s (1995) and Lalumière et al. (1995), renamed by Surbey and Brice (2007). Composed of 10 items, the questionnaire measures participant’s self-perceived mating popularity and attractiveness relative to their peers. Psychometric testing of the items has previously yielded good reliability (alpha = 0.87) (Lalumière and Quinsey 1996). Previous findings indicate that SPMV varies as a function of conditions or manipulations (Bird et al. 2016; Landolt et al. 1995: Surbey and Brice 2007). Sample items include “Members of the opposite sex notice me” and “Members of the opposite sex are attracted to me.” Participants were instructed to indicate, on a 7-point Likert scale, the extent to which the statements applied to them. Following reversal of applicable items, responses were totaled so that higher scores indicated higher SPMV.

#### Rosenberg Self-Esteem Scale

This widely used global measure of self-esteem (Rosenberg 1965) consists of 10 items to which participants responded using a 4-point scale. A sample item is “I take a positive attitude towards myself.” High scores indicate high levels of self-esteem. The measure has high test-retest reliability, with Cronbach’s alpha estimated between 0.77 and 0.88 across several samples (O’Brien 1985). Although baseline scores exhibit individual differences, they are also subject to temporary or situational fluctuations tapped by repeated measurement (Kernis et al. 1992; Rosenberg 1986).

#### Revised Sociosexual Orientation Inventory (SOI-R)

Sociosexuality refers to the level of endorsement of, or willingness to engage in, unrestricted sexual relations without closeness and commitment. The SOI-R (Penke and Asendorpf 2008) is a revision of Simpson and Gangestad’s (1991) original SOI, which captured individual differences in sociosexuality. The SOI-R is a nine-item scale measuring three facets of sociosexuality that can be examined conjointly or separately: Attitude, Desire, Behavior. The Attitude facet indicates endorsement of short-term, uncommitted sexual relationships and was expected to vary as a result of the manipulation, as did similar items of the original SOI employed by Surbey and Brice (2007). A sample item is “Sex without love is OK.” The Desire facet indicates participants’ desire for short-term relationships and was expected to be correlated with the Attitude facet and vary in a similar way. A sample item is “How often do you have fantasies about having sex with someone with whom you are not in a committed romantic relationship?” The Behavior facet measures cumulative number of sexual partners and similar characteristics; because the sample was relatively young and the time frame for the study relatively short, it was not expected to change over the experiment. High scores indicated higher levels of sociosexuality.

#### Approach Scale

This scale measured participants’ likelihood of approaching different people at a hypothetical post-race get together, a common event in the rowing community. As a behavioral measure of participants’ mating effort, it included three items measuring participant’s intentions to approach attractive women with whom the participant considered having a sexual relationship or dating. A sample individual was “a very attractive woman with whom you would consider having a sexual relationship.” The scale also contained three items involving the participant approaching people leading to their involvement with children or childcare (a proxy of parental effort). An example of this type of individual was “a recently widowed member of your extended family who you know is looking for a male mentor for her young son.” The classic findings that people’s intentions are reliable predictors of future behaviors (Eagly and Chaiken 1993; Fishbein and Ajzen 1975) provide validation for the use of measures of behavioral intentions. In addition to the six target items the scale included the possibility of approaching nine other individuals, such as “a person who is well connected and might be able to help you get an interview for a good job” or “a high profile athlete who might be able to give you some pointers regarding your sport” to reduce participants’ awareness of, or distract them from, the true intention of the measure. Before indicating their anticipated likelihood of approaching all hypothetical individuals, participants were asked to “Assume that you are single and looking forward to building a successful and interesting life for yourself” as a means of eliminating any effects of relationship status and providing a rational for the inclusion of the various items. A 7-point response scale was employed, with high total scores on each set of target items indicating a heightened likelihood of approaching attractive women (Woman Approach) or individuals with whom involvement with children would result (Child Involvement). To determine a potential trade-off between mating and parenting effort (M-P Trade-off), Child Involvement scores were subtracted from Woman Approach scores to produce a composite measure.

### Hormone Assays

The saliva samples were analyzed for testosterone concentrations using enzyme immunoassay kits purchased from Cambridge Biosciences (Cambridge, UK). The plates were coated with antibodies to testosterone. Samples were assayed in duplicate. (For further details of the immunoassay technique see Granger et al. 1999.) Intra-assay coefficients of variation (CV) were 4.6 and 4.3%, with an inter-assay coefficient of variation of 4.5%. Baseline testosterone levels were within the normal range (*M* = 60.21 pg/mL, *SD* = 6.09).

### Statistical Analyses

Analyses were conducted with the Statistical Package for the Social Sciences (SPSS), v.20, employing α = .05. Overall pre-race to post-race changes were examined via mixed between-within ANOVAs. Specific a priori predictions were tested using *t* tests based on the MSerror and degrees of freedom from the omnibus results (Howell 2013) with effect size, Cohen’s *d*, based on means and standard deviations. Although a priori predictions were directional, planned comparisons were conducted with two-tailed tests. These are more conservative but increase Type II errors, so this should be kept in mind when evaluating marginal results. Correlations were evaluated using Pearson’s Product Moment Correlations. Multiple regression analyses were employed to further elucidate the relationships between potential predictors and outcome measures.

## Results

### Preliminary Analyses

Since participants were randomly assigned a win or a loss, we did not expect to observe any differences between winners and losers in their pre-race testosterone, SPMV, Woman Approach, Child Involvement, M-P Trade-off, SOI-R Total, SOI-R Attitude, SOI-R Behavior, SOI-R Desire or Self-esteem. To confirm this was we conducted independent samples *t* tests on each variable and found no significant differences between winners and losers (see below). Furthermore, the randomly assigned winners and losers did not differ by age or 2000 m personal best time.

The demographic variables (age and relationship status) were analyzed for relationships with the dependent measures (Woman Approach, Child Involvement, and M-P Trade-off) or as possible confounds. Age was not significantly correlated with any of the dependents, except with pre-race self-esteem, where a positive association was found (*r* = .34, *n* = 38, *p* = .039). A series of *t* tests showed relationship status did not differ in the win/lose conditions, nor was it related to levels of the key variables in pre- or post-race conditions. Nonetheless all key analyses were run controlling for age and relationship status, but neither variable was found to be a significant factor in any of the results. Therefore, they were ruled out as possible confounders and not included in the final analyses. Intercorrelations for each set of three items totaled to produce Woman Approach and Child Involvement scores were highly significant (all Woman Approach *p* < .001, all Child Involvement *p* < .05). The data were also checked for errors, outliers, and violations of assumptions (normality, linearity, homogeneity of variance, homoscedascity, sphericity), but none were apparent. The means and ranges of pre-race testosterone and the psychological measures and comparisons of subsequent winners and losers on these measures are presented in Table 1.

**Table 1:** Descriptive statistics for pre-race testosterone and psychological measures, with independent *t* test (df = 36) comparisons of subsequent winners and losers

	Total Sample (*N* = 38)		Winners (*n* = 19)		Losers (*n* = 19)		Comparison of winners and losers	
	*M*	(*SD*)	*M*	(*SD*)	*M*	(*SD*)	*t*	*p*
Age	20.74	(1.20)	20.63	(1.39)	20.84	(1.01)	0.54	0.60
2000 m personal best (s)	400.29	(7.42)	401.53	(7.45)	400.05	(1.74)	0.19	0.85
Pre-race testosterone (pg/ml)	60.21	(6.09)	60.75	(5.72)	59.66	(6.54)	0.55	0.59
Pre-race SPMV	45.95	(5.30)	46.53	(4.88)	45.37	(5.76)	0.67	0.51
Pre-race Woman Approach	14.32	(2.59)	14.37	(2.27)	14.26	(2.94)	0.12	0.90
Pre-race Child Involvement	14.39	(1.22)	14.42	(1.39)	14.37	(1.07)	0.13	0.90
Pre-race M-P Trade-off	−0.08	(3.11)	−0.05	(2.57)	−0.11	(3.63)	0.05	0.96
Pre-race SOI-R Total	29.29	(6.80)	29.95	(5.81)	28.63	(7.78)	0.59	0.56
Pre-race SOI-R Attitude	13.42	(3.94)	13.95	(3.49)	12.89	(4.37)	0.82	0.42
Pre-race SOI-R Behavior	3.18	(2.26)	2.79	(1.72)	3.58	(2.69)	1.08	0.29
Pre-race SOI-R Desire	12.68	(3.02)	13.21	(3.03)	12.16	(3.01)	1.08	0.29
Pre-race self-esteem	18.00	(1.47)	17.68	(1.16)	18.32	(1.70)	1.34	0.19

Key: SPMV = self-perceived mate value; M-P Trade-off = mating-parenting trade-off; SOI-R = Revised Sociosexual Orientation Inventory

### Relationships between Baseline Testosterone and Psychological/Behavioral Variables

As expected, pre-race testosterone was significantly positively correlated with baseline SPMV, Woman Approach, M-P Trade-off, SOI-R Total, SOI-R Attitude, and SOI-R Desire. There were no significant correlations between pre-race testosterone and SOI-R Behavior or Self-esteem. Pre-race SPMV was related in a similar fashion to these variables, but the correlation with SOI-R Attitude did not achieve significance (Table 2). Although the sample size (*n* = 38) was limited, the correlation coefficients were moderate to large and replicated previous findings (e.g., Goodwin et al. 2012; Surbey and Brice 2007). Replications with additional and larger samples would further corroborate such reported baseline relationships.

**Table 2:** Correlation matrix of pre-race testosterone and psychological measures (*N* = 38)

						SOI-R				
	T (pg/ml)	SPMV	Woman approach	Child	M-P Trade-off	Total	Attitude	Behavior	Desire	Self-esteem
Testosterone (pg/ml)	–									
SPMV	.78**	–								
Woman approach	.33*	.35*	–							
Child involvement	−.45**	−.47**	−.30	–						
M-P Trade-off	.45**	.47**	.92***	−.58***	–					
SOI-R Total	.39*	.35*	.23	−.29	.30	–				
SOI-R Attitude	.35*	.25	.10	−.22	.17	.92***	–			
SOI-R Behavior	.18	.23	.48**	−.14	.46**	.38*	.17	–		
SOI-R Desire	.29	.30	.02	−.27	.12	.77***	.63***	−.11	–	
Self-esteem	−.10	−.15	−.07	−.12	−.01	−.00	.03	−.11	.03	–

SPMV, self-perceived mate value; M-P Trade-off = mating-parenting trade-off; SOI-R, Revised Sociosexual Orientation Inventory

*** *p* < 0.001, ** *p* < 0.01, * *p* < 0.05

### Two-Way Mixed ANOVAs Comparing Winners with Losers over Time

**Fig. 1:**
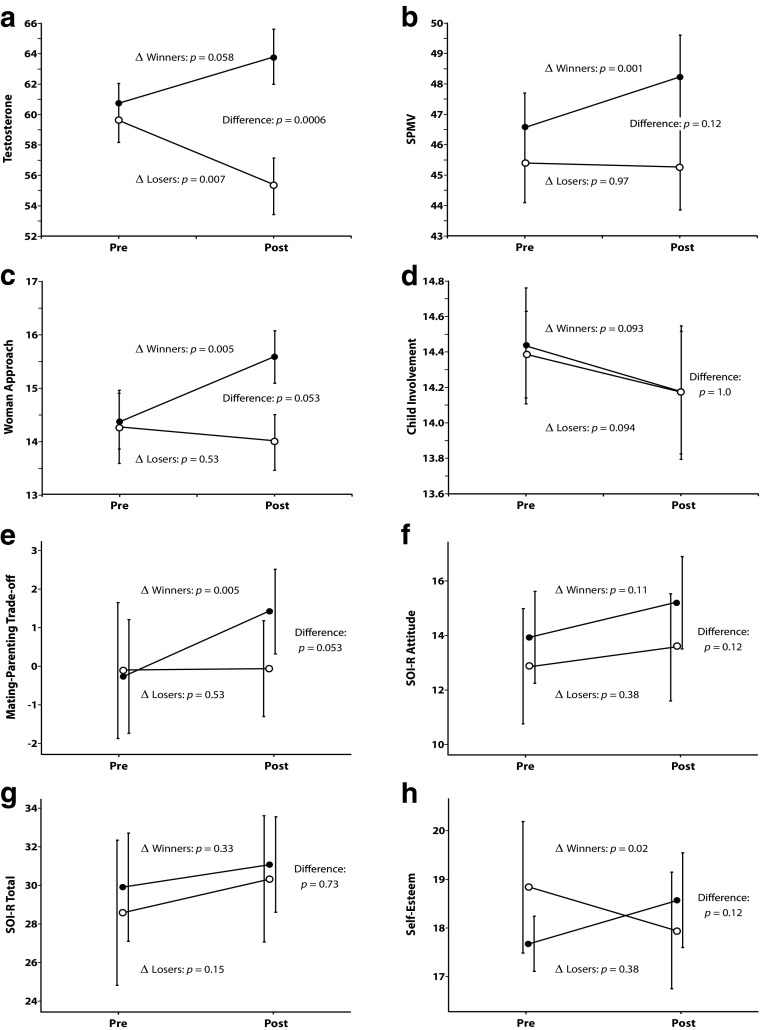
Response to competition outcome. ● = Winners, ○ = Losers. Standard error bars are shown

Two-way mixed ANOVAs were conducted to compare differences in testosterone and other measures between winners and losers as well as over time (before and after the race). Significant interactions were found for testosterone, SPMV, Woman Approach, and M-P Trade-off (Table 3). The interactions and main effects for all outcome measures are plotted in Fig. 1.

**Table 3:** Two-way mixed ANOVAs to compare differences in testosterone and other measures between winners and losers, and over time

Variable	*F*_1,36_	η_*p*_^2^	*p*
Testosterone			
Time	.38	–	.54
Race result	5.52	.24	.024
Time*Race result	11.45	.24	.002
SPMV			
Time	4.92	.12	.033
Race result	1.30	–	.26
Time*Race result	7.26	.17	.011
Woman Approach			
Time	2.72	–	.11
Race result	1.29	–	.26
Time*Race result	6.58	.16	.015
Child Involvement			
Time	.46	–	.50
Race result	.01	–	.996
Time*Race result	.01	–	.94
M-P Trade-off			
Time	3.89	–	.056
Race result	.89	–	.35
Time*Race result	4.48	.11	.041
SOI-R Total			
Time	3.04	–	.09
Race result	.29	–	.60
Time*Race result	.12	–	.73
SOI-R Attitude			
Time	3.17	–	.084
Race result	1.42	–	.24
Time*Race result	.28	–	.60
SOI-R Desire			
Time	.47	–	.50
Race result	.51	–	.48
Time*Race result	1.73	–	.20
Rosenberg Self-Esteem			
Time	.99	–	.33
Race result	.00	–	1.00
Time*Race result	.567	.14	.023

### Planned Comparisons of A Priori Predictions Regarding the Effects of the Manipulated Win and Loss over Time

Planned comparisons were conducted to test the a priori predictions regarding the effects of the manipulated win and loss over time.

#### Testosterone

The testosterone levels of losers significantly declined following the race (*M* = 55.34, *SD* = 8.01) compared with pre-race values (*M* = 59.66, *SD* = 6.54; *t*_36_ = 2.83, *p* = .007, *d* = .59). Conversely, testosterone levels of winners were marginally significantly higher post-race (*M* = 63.74, *SD* = 7.74) than pre-race (*M* = 60.75, *SD* = 5.72; *t*_36_ = 1.31, *p* = .058, *d* = .44). These changes resulted in the post-race testosterone levels of winners being significantly higher than those of losers (*t*_*5*5_ = 3.67, *p* = .0006, *d* = 1.07).

#### SPMV

Following the race the SPMV of winners significantly increased from pre-race values (before: *M* = 46.53, *SD* = 4.88; after: *M* = 48.16, *SD* = 5.78, *t*_36_ = 3.47, *p* = .001, *d* = .31), whereas the SPMV of losers did not change (before: *M* = 45.37, *SD* = 5.76; after: *M* = 45.21, *SD* = 6.05; *t*_36_ = 0.34, *p* = .97). The post-race SPMV levels of winners were not, however, significantly different from those of losers *(t*_55_ = 1.61, *p* = .12).

#### Woman Approach

The Woman Approach scores of winners significantly increased from pre- to post-race values (before: *M* = 14.37, *SD* = 2.27; after: *M* = 15.58, *SD* = 2.14; *t*_36_ = 2.99, *p* = .005, *d* = .55), whereas those of losers did not change (before: *M* = 14.26, *SD* = 2.94; after: *M* = 14.00, *SD* = 2.38; *t*_36_ = 0.64, *p* = .53). This resulted in the post-race Woman Approach scores of winners being borderline significantly greater than those of losers (*t*_55_ = 1.99, *p* = .053, *d* = .11).

#### Child Involvement

Child Involvement scores of winners (before: *M* = 14.42, *SD* = 1.39; after: *M* = 14.16, *SD* = 1.61) and losers (before: *M* = 14.37, *SD* = 1.07; after: *M* = 14.16, *SD* = 1.46) did not change from pre-race values (Winners *t*_18_ = .09, *p* = .93, *d* = .17; Losers *t*_18_ = .07, *p* = .94). Consequently, there was no significant difference between the post-race Child Involvement of winners and losers (*t*_69_ = 0.00, *p* = 1.00).

#### M-P Trade-Off (Woman Approach − Child Involvement)

The M-P Trade-off of winners significantly increased from pre- to post-race values (before: *M* = −0.05, *SD* = 2.57; after: *M* = 1.42, *SD* = 2.27; *t*_18_ = 2.89, *p* = .006, *d* = .61), whereas the trade-off of losers did not change (before: *M* = −0.105, *SD* = 3.63; after: *M* = −0.16, *SD* = 2.87; *t*_18_ = 0.10, *p* = .92). As a result, the difference in the post-race trade-off of winners and losers approached, but did not achieve, significance (*t*_48_ = 1.69, *p* = .098).

#### SOI-R Total

The SOI-R Total of neither winners (before: *M* = 29.95, *SD* = 5.81; after: *M* = 31.11, *SD* = 5.10; *t*_18_ = .99, *p* = .33) nor losers (before: *M* = 28.63, *SD* = 7.78; after: *M* = 30.37, *SD* = 6.75; *t*_18_ = 1.48, *p* = .15) changed. Consequently, there was no difference between the post-race SOI-R Total of winners and losers (*t*_49_ = 0.35, *p* = .73).

#### SOI-R Attitude

The SOI-R Attitude of winners increased after the race as expected, but not significantly (before: *M* = 13.95, *SD* = 3.49; after: *M* = 15.21, *SD* = 3.49; *t*_18_ = 1.62, *p* = .11). Losers’ scores also did not significantly change pre- and post-race (before: *M* = 12.89, *SD* = 4.37; after: *M* = 13.58, *SD* = 4.03, *t*_18_ = 0.89, *p* = .38). Consequently, the post-race SOI-R Attitude of winners was not significantly different than that of losers (*t*_52_ = 1.30, *p* = .12).

#### SOI-R Desire

Following the race the SOI-R Desire of neither winners nor losers changed (Winners before: *M* = 13.21, *SD* = 3.03; after: *M* = 12.95, *SD* = 1.93; *t*_18_ = 0.44, *p* = .67; Losers before: *M* = 12.16, *SD* = 3.01; after: *M* = 13.00, *SD* = 1.86, *t*_18_ = 1.41, *p* = .17). Consequently, there was no significant difference between the post-race SOI-R Desire of winners and losers (*t*_59_ = 0.06, *p* = .95).

#### SOI-R Behavior

Since there was a relatively short time between the pre- and post-race measurements, none of the participants changed any of their answers on the behavior section of the SOI-R questionnaire. As anticipated, this was not a relevant subscale for further examination.

#### Rosenberg Self-Esteem Scale

Whereas the self-esteem of winners significantly increased from pre-race values (before: *M* = 17.68, *SD* = 1.16; after: *M* = 18.58, *SD* = 2.01; *t*_18_ = 2.40, *p* = .022, *d* = .55), the self-esteem of losers did not change (before: *M* = 18.32, *SD* = 1.70; after: *M* = 17.95, *SD* = 2.46, *t*_18_ = 0.99, *p* = .33). No significant difference between the post-race self-esteem of winners and losers was found (*t*_52_ = 1.03, *p* = .30).

### The Role of Changes in Testosterone Following a Manipulated Win or Loss

The previous results show that testosterone significantly declined in losers and increased in winners following the race. In addition, SPMV, Woman Approach, and Mating-Parenting Trade-off significantly increased in winners relative to losers. Whether such post-manipulation differences were attributable to the contrasting direction of change in testosterone in the two groups was examined next. Separate hierarchical multiple regressions were conducted on the change (Δ) in SPMV, Woman Approach, Child Involvement, Mating-Parenting Trade-off and SOI-R Total to examine the main effects of ΔT, and the manipulation (Race Result), and their interaction (ΔT × Race Result). Changes in variables were calculated by subtracting pre-race values from post-race values. A significant interaction indicated that the opposite change in testosterone levels in the two groups (increase in winners and decrease in losers) significantly predicted the observed post-race changes in the outcome measures in winners versus losers.

Table 4 shows that the interaction (or the differing change in testosterone levels in the two groups following the manipulation) significantly predicted the ΔSPMV in each group. Together, the predictors accounted for 22.9% of the variance in ΔSPMV. The predictors explained 13.5% of variation in ΔWoman approach, 5.5% of variation in ΔChild involvement, 20.7% of variation in ΔMating-parenting trade-off and 1.3% of variation of ΔSOI-R Total.

**Table 4:** Summary of the role of contrasting changes in Testosterone (T) in Winners and Losers (ΔT × Race Result)^†^ in changes in outcome measures following the manipulation

Outcome measure	Predictor	*Β*	*t*	*P*
ΔSPMV	ΔT	−.005	−.097	.923
	Race result	1.092	1.480	.148
	ΔT × Race result	.246	2.165	.038
	Model Summary: *F*_3,34_ = 4.653, *p =* 0.008*, R*^2^ = .291, *adj. R*^2^ = .229			
ΔWoman approach	ΔT	−.021	−.419	.678
	Race result	1.185	1.769	.086
	ΔT × Race result	.148	1.435	.160
	Model Summary: *F*_3,34_ = 2.926, *p =* 0.048*, R*^2^ = .205, *adj. R*^2^ = .135			
ΔChild involvement	ΔT	.013	.309	.759
	Race result	.126	.219	.828
	ΔT × Race result	−.180	−2.034	.050
	Model Summary: *F*_3,34_ = 1.725, *p =* 0.180*, R*^2^ = .132, *adj. R*^2^ = .055			
ΔMating-parenting trade-off	ΔT	−.033	−.550	.586
	Race result	.820	1.044	.304
	ΔT × Race result	.316	2.609	.013
	Model Summary: *F*_3,34_ = 4.228, *p =* 0.012*, R*^2^ = .272, *adj. R*^2^ = .207			
ΔSOI-R Total	ΔT	.191	1.309	.199
	Race result	−2.047	−1.061	.296
	ΔT × Race result	.023	.078	.938
	Model Summary: *F*_3,34_ = .841, *p =* 0.481*, R*^2^ = .069, *adj. R*^2^ = −.013			

† includes the final step in separate hierarchical multiple regression analyses showing the main effects of Δ Testosterone and Race Result, and their interaction on changes in the outcome measures

### Intercorrelations between Post-Race Scores Following the Manipulation

Please see the ESM for a table that provides the correlations between post-race variables, as well as variable means and standard deviations (on the diagonal). As expected the post-race values of testosterone and SPMV were significantly and positively correlated with each other and with post-race Woman Approach and M-P Trade-off scores, and some components of SOI-R. Self-esteem was only significantly correlated with Child Involvement and consequently M-P Trade-off, whereby men with high self-esteem indicated that they would be less likely to become involved with children relative to approaching attractive women (without children). The next question to be addressed was which variables best predicted post-race outcome scores and if there was any evidence that the psychological variables played a mediational role in the effects of testosterone.

### Best Predictors of Post-Race Scores and Potential Mediation of the Effects of Testosterone

Hierarchical multiple regression analysis was performed to investigate whether post-race testosterone or the psychological variables SPMV and self-esteem best or independently predicted the post-race values of Woman Approach, Child Involvement, M-P Trade-off, and SOI-R Total. Whether there was any evidence of mediation of the effects of testosterone by SPMV was also examined. To test for mediation effects we employed the method of Baron and Kenny (1986). With the exception of Child Involvement, testosterone and SPMV were significantly correlated to each other and the dependent variables, satisfying the first two criteria of Baron and Kenney for the remaining variables. For these variabless, two regression models were considered. In the first model, testosterone was added on the first step, allowing the effects of SPMV to be considered after controlling for the effect of testosterone. If SPMV was no longer a significant predictor when entered in the second step, this would suggest its effect was subsumed by testosterone. In the second model, SPMV was added first, allowing the effects of testosterone to be considered on the second step, after accounting for SPMV. In this case, SPMV would be shown to be a potential mediator in the event the effect of testosterone was reduced to non-significance. In both models, self-esteem was added in the third step to determine if it explained any further variance beyond that explained by SPMV.

The findings suggest that while high levels of both testosterone and SPMV predicted higher Woman Approach scores on their own, SPMV appeared to mediate the effects of testosterone. Self-esteem did not contribute significantly to the model, and together the variables accounted for 41% of the variance in Woman Approach scores (Table 5).

**Table 5:** Hierarchical regression. Alternative models for the relationship between predictors and Woman Approach

Step	Predictor	*Β*	*t*	*P*
	Model 1			
1	Testosterone	.57	4.20	<.001
	Δ *R*^2^ = .32, *F* Change _1,36_ = 17.65, *p* < .001			
1	Testosterone	.31	1.90	.065
2	SPMV	.43	2.68	.011
	Δ *R*^2^ = .11, *F* Change _1,35_ = 7.20, *p* = .01			
1	Testosterone	.31	1.93	.062
2	SPMV	.42	2.59	.014
3	Self-esteem	.11	.86	.399
	Δ *R*^2^ = .01, *F* Change _1,34_ = .73, *p* = .40			
	Model 2			
1	SPMV	.62	4.76	<.001
	Δ *R*^2^ = .39, *F* Change _1,36_ = 22.62, *p* < .001			
1	SPMV	.43	2.68	.011
2	Testosterone	.31	1.90	.065
	Δ *R*^2^ = .06, *F* Change _1,35_ = 3.62, *p* = .07			
1	SPMV	.42	2.59	.014
2	Testosterone	.31	1.93	.062
3	Self-esteem	.11	.86	.399
	Δ *R*^2^ = .01, *F* Change _1,34_ = .73, *p* = .40			

Model Summary: *F*_3,34_ = 9.65, *p* < 0.001, *R*^2^ = .46, *adj. R*^2^ = .41

SPMV = self-perceived mate value

Outcomes of the hierarchical regressions predicting the difference in the likelihood of men approaching attractive women versus individuals who would lead to involvement with children, as indicated by M-P Trade-off, are given in Table 6. The findings suggest that while initially high levels of testosterone and SPMV independently predicted M-P Trade-off, SPMV appeared to mediate the effect of testosterone, and self-esteem contributed further and significantly to the model. Together the variables accounted for 39% of the variance in M-P Trade-off scores.

**Table 6:** Hierarchical regression. Alternative models for the relationship between predictors and M-P Trade-off

Step	Predictor	*Β*	*t*	*P*
	Model 1			
1	Testosterone	.50	3.47	.001
	Δ *R*^2^ = .25, *F* Change _1,36_ = 12.04, *p* = .001			
1	Testosterone	.28	1.59	.122
2	SPMV	.36	2.01	.052
	Δ *R*^2^ = .078, *F* Change _1,35_ = 4.05, *p* = .05			
1	Testosterone	.30	1.82	.078
2	SPMV	.32	1.95	.059
3	Self-esteem	.34	2.62	.013
	Δ *R*^2^ = .11, *F* Change _1,34_ = 6.87, *p* = .01			
	Model 2			
1	SPMV	.53	3.74	.001
	Δ *R*^2^ = .28, *F* Change _1,36_ = 14.01, *p* = .001			
1	SPMV	.36	2.01	.052
2	Testosterone	.28	1.59	.122
	Δ *R*^2^ = .05, *F* Change _1,35_ = 2.51, *p* = .12			
1	SPMV	.32	1.95	.059
2	Testosterone	.30	1.82	.078
3	Self-esteem	.34	2.62	.013
	Δ *R*^2^ = .11, *F* Change _1,34_ = 6.87, *p* = .01			

Model Summary: *F*_3,34_ = 8.95, *p* < 0.001, *R*^2^ = .44, *adj. R*^2^ = .39

M-P Trade-off = mating-parenting trade-off; SPMV = self-perceived mate value

Outcomes of the hierarchical regressions predicting men’s overall endorsement of short-term strategies, as measured by the SOI-R Total, are given in Table 7. The findings suggest that while high levels of testosterone and SPMV independently predicted SOI-R Total, SPMV was not a significant predictor when both variables were entered. Self-esteem did not contribute significantly to the model, and together the variables accounted for 22% of the variance in SOI-R Total scores.

**Table 7:** Hierarchical regression. Alternative models for the relationship between predictors and SOI-R Total

Step	Predictor	*Β*	*t*	*P*
	Model 1			
1	Testosterone	.50	3.45	.001
	Δ *R*^2^ = .25, *F* Change _1,36_ = 11.88, *p* = .001			
1	Testosterone	.48	2.59	.014
2	SPMV	.02	.12	.903
	Δ *R*^2^ = .00, *F* Change _1,35_ = .02, *p* = .90			
1	Testosterone	.49	2.66	.012
2	SPMV	.00	.02	.985
3	Self-esteem	.19	1.27	.214
	Δ *R*^2^ = .03, *F* Change _1,34_ = 1.61, *p* = .21			
	Model 2			
1	SPMV	.32	2.05	.048
	Δ *R*^2^ = .10, *F* Change _1,36_ = 4.20, *p* = .048			
1	SPMV	.02	.12	.903
2	Testosterone	.48	2.59	.014
	Δ *R*^2^ = .14, *F* Change _1,35_ = 6.71, *p* = .01			
1	SPMV	.00	.02	.985
2	Testosterone	.49	2.66	.012
3	Self-esteem	.19	.13	.214
	Δ *R*^2^ = .03, *F* Change _1,34_ = 1.61, *p* = .21			

Model Summary: *F*_3,34_ = 4.46, *p* = 0.01, *R*^2^ = .28, *adj. R*^2^ = .22

SOI-R = Revised Sociosexual Orientation Inventory; SPMV = self-perceived mate value

## Discussion

We examined relationships among men’s naturally occurring (pre-race) testosterone, their self-perceived mate value, self-esteem, sociosexuality, and anticipated likelihood of approaching attractive women versus situations leading to involvement with children. We then monitored changes in these measures following a manipulated win or loss as a result of an indoor rowing contest. Baseline (pre-race) results revealed that both heightened testosterone and SPMV were associated with a greater orientation toward engaging in casual sexual relationships and a higher likelihood of initiating relationships with attractive women in a hypothetical social situation. This is consistent with previous work suggesting a link between androgenization and sexual desire (Baumeister et al. 2001), as well as greater levels of sexual activity (Bogaert and Fisher 1995; Oltmanns et al. 2008). In relation to SPMV, the current findings are consistent with previous reports linking men’s assessments of their own mate value to mating strategy (Surbey and Brice 2007). Furthermore, we observed that before the race both high testosterone and SPMV were associated with a reduced inclination to become involved with or mentor children. Moreover, there was a negative relationship between Woman Approach and Child Involvement that approached significance (*p* = .08, one-tailed), a condition suggestive of a trade-off (Roff 1992; Stearns 1992). When considered in concert with the literature relating high testosterone and SPMV levels with increased mating effort and concurrently decreased parenting effort, it would appear that endocrine and psychological systems work in tandem to regulate this male reproductive trade-off (e.g., Alvergne et al. 2009; Berg and Wynne-Edwards 2001; Fleming et al. 2002; Gettler et al. 2011b; Gray et al. 2004a, 2007b; Kuzawa et al. 2009; McGlothlin et al. 2007; McIntyre et al. 2006; Muller et al. 2009; Perini et al. 2012; Pollet et al. 2013; Storey et al. 2000).

In many species, male status in a social hierarchy emerges through male-male competition. A considerable number of previous researchers have reported a link between competition outcome and androgenization in human males, with a victory typically causing an increase in testosterone relative to defeat (Archer 2006; Bernhardt and Dabbs 1997; Bernhardt et al. 1998; Booth et al. 1989; Elias 1981; Gladue et al. 1989; Mazur et al. 1992; Mazur and Lamb 1980). The findings of the present study are consistent with previous reports as perceived victory in the ergometer competition led to an increase in testosterone, while defeat led to a decrease. However, in the past it was not known if this was driven by a stable association between physical power and testosterone (i.e., winners may be stronger and have higher baseline testosterone levels) or whether testosterone levels are responsive in the short-term to the social experience of winning. The manipulated competition outcome used in this investigation suggests that the social experience of winning significantly contributes to the surge in androgenization and associated psychological changes. This investigation further extends previous studies by considering the effect of competition outcome on psychological measures relevant to reproductive effort, in addition to endocrine responses. These findings have important implications for male reproductive function since investment in both physiological and behavioral effort is required for reproductive success.

The rowing competition led to an increase in testosterone, SPMV, Woman Approach, M-P Trade-off and self-esteem in purported winners relative to losers, whose levels typically declined or remained the same. Furthermore, winners exhibited a tendency toward increased sociosexuality, a measure of engaging in casual, uncommitted sexual relationships. Moreover, the contrasting changes in testosterone in winners and losers significantly predicted the changes in SPMV, Child Involvement, and M-P Trade-off in the groups following the manipulation. Both post-race testosterone and SPMV levels were significant correlates of the post-race outcome measures, whereas self-esteem was only correlated with men’s likelihood of becoming involved with children, and consequently with the M-P Trade-off.

Hierarchical multiple regressions were conducted to determine if testosterone, SPMV, and self-esteem independently predicted the variance in outcome measures and if there was any evidence that SPMV or self-esteem mediated the effects of testosterone. Earlier we proposed that psychological factors may mediate the effects of testosterone on mating-related behavior. That is, that changes in testosterone precipitate psychological processes that in turn underlie alterations in mating behavior. Results revealed that while both testosterone and SPMV, but not self-esteem, significantly predicted men’s post-race likelihood of approaching attractive women, adding SPMV after testosterone in the model reduced the effects of testosterone to non-significance. Thus SPMV appeared to mediate the effects of testosterone in men’s likelihood of approaching attractive women at a hypothetical rowing after-party. Generally, self-esteem was not a significant correlate of the other measures. An exception was that it was the only significant predictor of men’s likelihood of involvement with children, with men with high self-esteem less likely to become so involved. This resulted in self-esteem also playing a role in predicting men’s bias (trade-off) toward approaching attractive women (a proxy for mating effort) versus Child Involvement (a proxy for parental effort). Both testosterone and SPMV independently predicted men’s bias, with SPMV apparently mediating the effects of testosterone. When both testosterone and SPMV were accounted for, self-esteem made an additional significant contribution to the model, with the three predictors together accounting for 39% of the variance in men’s M-P Trade-off. Therefore men with high levels of testosterone, SPMV, and self-esteem following the race exhibited a heightened likelihood of approaching attractive women versus becoming involved with children. Following the manipulation, both testosterone and SPMV were significantly and positively correlated with total score on the SOI-R, a measure of sociosexuality. Hierarchical multiple regression suggested, however, that SPMV or self-esteem were not significant predictors of sociosexuality after accounting for the effects of testosterone.

**Fig. 2:**
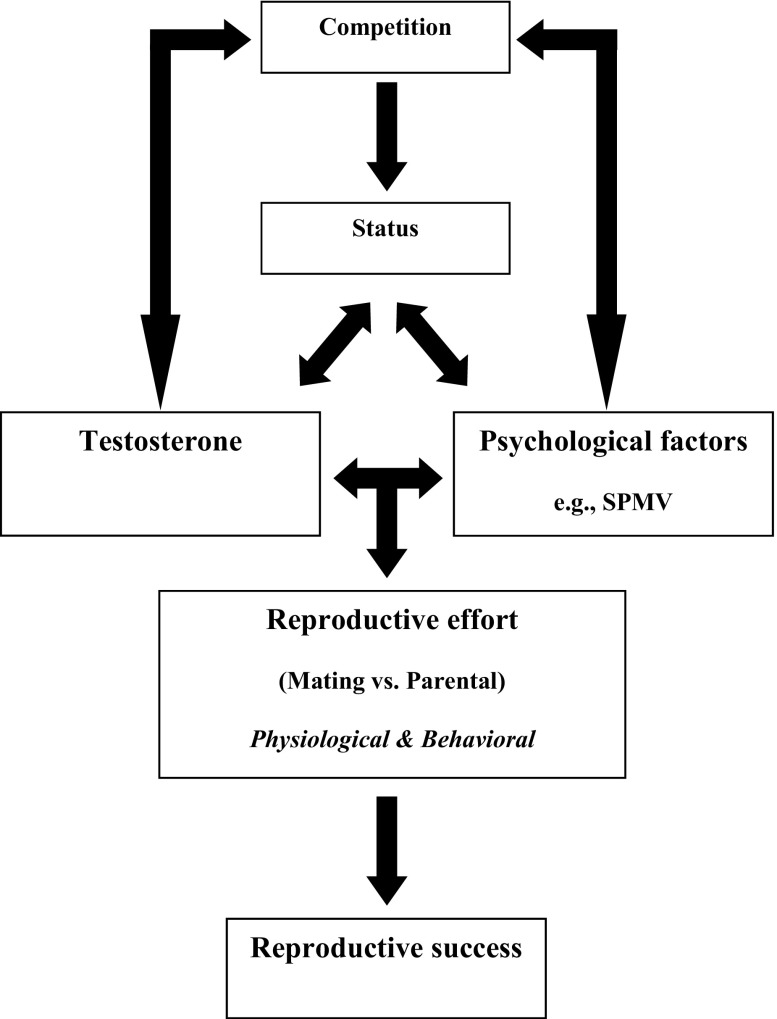
Proposed model for androgenic and psychological influences on male reproductive effort

That SPMV appeared to mediate some of the effects of testosterone complements the reciprocal model of testosterone and status (Mazur and Booth 1998) and provides further evidence of a pathway between endocrinological responses and changes in mating- and parent-related behavior. We propose a broader model in which SPMV is one psychological factor mediating the effects of testosterone in enhancing male reproductive effort. In this model, depicted in Fig. 2, reciprocal relationships exist between competition, status, testosterone, psychological factors, and behaviors relevant to reproductive success.

Our findings suggest a concurrent physiological and psychological dynamism in individual investment in mating and parental effort not captured with previous methodologies. Although body size, shape, and endocrine profiles presumably vary somewhat genetically, our results show that both testosterone and its psychological correlates fluctuate opportunistically and predictably. We conducted the assays on afternoon testosterone levels, which reportedly track activities and short-term socioenvironmental factors, but morning testosterone levels are a better indication of endogenous physiological differences (Gray et al. 2007a). It would be interesting to compare men’s morning testosterone levels with afternoon fluctuations as a result of manipulated wins and losses both to control for them and to determine if some men (or genotypes) are more susceptible to such socioenvironmental influences. For example, men with predispositions for low testosterone levels may respond differently to a testosterone-boosting win than men with high inherent testosterone levels. Furthermore, one might expect the mechanisms involved in allocating resources to reproduction to be responsive to the outcome of multiple events rather than a single event, as demonstrated in this study. Further work employing a longitudinal design spanning multiple competitions could be better suited to consider the physiological and psychological response to contest outcome.

Although this study has shown an endocrine response to a sporting contest, several studies have reported similar findings in non-athletic contests, such as chess (Mazur et al. 1992; Mazur and Lamb 1980), video-gaming (Zilioli and Watson 2012), and laboratory-based reaction-time contests (Gladue et al. 1989). Future research should perhaps aim to further investigate the physiological and psychological consequences of victory and defeat in non-athletic contests. This is because competition of a non-athletic nature is perhaps the more salient mode of rivalry in contemporary Western society. Our sample of participants was reasonably small and specialized. Replication of the findings reported here in diverse populations employing other forms of competition would demonstrate their generalizability.

To conclude, this investigation has demonstrated an increase in androgenization, SPMV, and a greater willingness to approach attractive women versus individuals leading to involvement with children as a result of perceived victory in a male-male sporting contest. Future avenues of research may apply a similar protocol to more intellectually based competitions.

## Electronic supplementary material

ESM 1 (PDF 138 kb)
