# Supplementary material for: Tandem Androgenic and Psychological Shifts in Male Reproductive Effort Following a Manipulated “Win” or “Loss” in a Sporting Competition
Source: Hum Nat. 2018 Aug 9;29(3):283–310. doi: 10.1007/s12110-018-9323-5 (PMC6132838; doi:10.1007/s12110-018-9323-5)
Supplement: Supplementary file 1 — (PDF 138 kb) [file 12110_2018_9323_MOESM1_ESM.pdf]

Electronic Supplementary Material
for

Tandem Androgenic and Psychological Shifts in Male Reproductive Effort Following a Manipulated "Win" or "Loss" in a Sporting Competition

D. Longman[1*], M. K. Surbey[2], J. T. Stock[3], and J. C. K. Wells[4]

[1] Department of Archaeology and Anthropology, University of Cambridge, Cambridge, CB2 3QG, UK, Email: dl329@cam.ac.uk

[2] Department of Psychology, James Cook University, Townsville, QLD, 4811, Australia

[3] Department of Anthropology, University of Western Ontario, Ontario, Canada

[4] Childhood Nutrition Research Centre, UCL Institute of Child Health, London, WC1N 1EH, UK

published in *Human Nature* 29(3), 2018, https://doi.org/10.1007/s12110-018-9323-5

Contents

1. Self-Perceived Mate Value Questionnaire
2. Self-esteem Scale
3. Revised Sociosexual Orientation Inventory (SOI-R)
4. Approach Scale
5. Correlation matrix of post-race testosterone and psychological measures

1. Self-Perceived Mate Value Questionnaire

For the following questions please rate your response on a scale of 1–7 by circling the appropriate number.

**Disagree**	1	2	3	4	5	6	7	**Agree**

1. Members of the opposite sex that I like tend to like me back. 1 2 3 4 5 6 7
2. Members of the opposite sex notice me. 1 2 3 4 5 6 7
3. I receive many compliments from members of the opposite sex. 1 2 3 4 5 6 7
4. Members of the opposite sex are not very attracted to me (R) 1 2 3 4 5 6 7
5. I receive sexual invitations from members of the opposite sex. 1 2 3 4 5 6 7
6. Members of the opposite sex are attracted to me. 1 2 3 4 5 6 7
7. I can have as many sexual partners as I choose. 1 2 3 4 5 6 7
8. I do not receive many compliments from members of the opposite sex. (R) 1 2 3 4 5 6 7

For the following questions please rate your response according to the scale below by circling the appropriate number.

9. Relative to my peer group, I can get dates:

**With great difficulty**	1	2	3	4	5	6	7	**With great east**

10. Relative to my peer group, I consider myself:

**Much less attractive**	1	2	3	4	5	6	7	**Much more attractive**

2. Self-Esteem Scale
Instructions: Below is a list of statements dealing with your general feelings about yourself. If you strongly agree, circle **SA**. If you agree with the statement, circle **A**. If you disagree, circle **D**. If you strongly disagree, circle **SD**.

1.	On the whole, I am satisfied with myself.	SA	A	D	SD
2.	At times, I think I am no good at all.	SA	A	D	SD
3.	I feel that I have a number of good qualities.	SA	A	D	SD
4.	I am able to do things as well as most other people.	SA	A	D	SD
5.	I feel I do not have much to be proud of.	SA	A	D	SD
6.	I certainly feel useless at times.	SA	A	D	SD
7.	I feel that I'm a person of worth, at least on an equal plane with others.	SA	A	D	SD
8.	I wish I could have more respect for myself.	SA	A	D	SD
9.	All in all, I am inclined to feel that I am a failure.	SA	A	D	SD
10.	I take a positive attitude toward myself.	SA	A	D	SD

3. The revised Sociosexual Orientation Inventory (SOI-R)

Please respond honestly to the following questions, ticking your answer:

1. With how many different partners have you had sex within the past 12 months?
- o 0
- o 1
- o 2
- o 3
- o 4
- o 5-6
- o 7-9
- o 10-19
- o 20 or more

2. With how many different partners have you had sexual intercourse on one and only one occasion?
- o 0

- 1
- 2
- 3
- 4
- 5-6
- 7-9
- 10-19
- 20 or more

3. With how many different partners have you had sexual intercourse without having an interest in a long-term committed relationship with this person?
   - 0
   - 1
   - 2
   - 3
   - 4
   - 5-6
   - 7-9
   - 10-19
   - 20 or more

4. Sex without love is OK.
   - 1 Strongly disagree
   - 2
   - 3
   - 4
   - 5
   - 6
   - 7
   - 8
   - 9 Strongly agree

5. I can imagine myself being comfortable and enjoying "casual" sex with different partners.
   - 1 Strongly disagree
   - 2
   - 3
   - 4
   - 5
   - 6
   - 7
   - 8
   - 9 Strongly agree

6. I do not want to have sex with a person until I am sure that we will have a long-term, serious relationship.
   - 1 Strongly disagree

- 2
- 3
- 4
- 5
- 6
- 7
- 8
- 9 Strongly agree

7. How often do you have fantasies about having sex with someone with whom you are not in a committed romantic relationship?

- 1 – never
- 2 – very seldom
- 3 – about once every two or three months
- 4 – about once a month
- 5 – about once every two weeks
- 6 – about once a week
- 7 – several times per week
- 8 – nearly every day
- 9 – at least once a day

8. How often do you experience sexual arousal when you are in contact with someone with whom you are not in a committed romantic relationship?

- 1 – never
- 2 – very seldom
- 3 – about once every two or three months
- 4 – about once a month
- 5 – about once every two weeks
- 6 – about once a week
- 7 – several times per week
- 8 – nearly every day
- 9 – at least once a day

9. In everyday life, how often do you have spontaneous fantasies about having sex with someone you have just met?

- 1 – never
- 2 – very seldom
- 3 – about once every two or three months
- 4 – about once a month
- 5 – about once every two weeks
- 6 – about once a week
- 7 – several times per week
- 8 – nearly every day
- 9 – at least once a day

4. Approach Scale

Imagine you are at a rowing competition after-party, and a number of other rowers, spectators, friends and families are there. Assume you are single and looking forward to building a successful and interesting life for yourself. Employing the scale below (by placing the appropriate number in the space before each item), indicate how likely you would be to approach the following people:

not at all likely 1 2 3 4 5 6 7 **very likely**

1. a **particularly attractive woman** you think you would like to go out with/date
2. a **person who is well connected** and might be able to help you get you an interview for a good job
3. **a person who has contacts** and might be able to get you a good deal on a fairly expensive consumer item you are interesting in buying
4. a **very attractive woman** with whom you would consider having a sexual relationship
5. a **well-known writer** whose book you recently read
6. a **local celebrity** you would enjoy meeting
7. a **high profile athlete** who might be able to give you some pointers regarding your sport
8. a **recently widowed female member of your extended family** who is looking for a male mentor for her young son
9. a **woman active in the community** looking for volunteers to run a local sports camp for young children
10. a **junior rowing team from a local club/school** who would probably enjoy meeting one of the more senior rowers in the race
11. a **divorced woman** with her three small children who would probably like to meet one of the rowers in the race
12. an **aged aunt and uncle** who often turn out to watch you race
13. a **very attractive male rower** you've heard has been offered a male modeling job as a result of his participation in a rowing team
14. the **quite attractive sister of** a **member of another team** you would like to talk with/get to know

Table ESM 1. Correlation matrix of post-race testosterone and psychological measures, showing mean (SD) scores (*N*=38).

						SOI-R				
	T (pg/ml)	SPMV	Woman Approach	Child Inv.	M-P Trade-off	Total	Attitude	Desire	Behavior	Self-esteem
Testosterone	59.55 (8.86)									
SPMV	.62***	46.69 (6.02)								
Woman Approach	.57***	.62***	14.79 (2.37)							
Child Involvement	.01	.04	.11	14.16 (1.52)						
M-P Trade-off	.50**	.53**	.84***	-.47**	.63 (2.68)					
SOI-R Total	.50**	.32*	.21	-.30	.35*	30.74 (5.91)				
SOI-R Attitude	.45**	.27	.11	-.25	.24	.87***	14.39 (3.81)			
SOI-R Desire	.41*	.21	.17	-.16	.23	.84***	.71***	12.97 (1.87)		
SOI-R Behavior	.24	.30	.25	-.18	.33*	.45**	.03	16	3.18 (2.26)	
Self-esteem	.01	.07	.14	-.42**	.36*	.19	.17	.13	.10	18.26 (2.24)

Key: *** $p < 0.001$, ** $p < 0.01$, * $p < 0.05$
SPMV = self-perceived mate value; M-P Trade-off = mating-parenting trade-off; SOI-R = Revised Sociosexual Orientation Inventory.
